# MiMics-Net: A Multimodal Interaction Network for Blastocyst Component Segmentation

**DOI:** 10.3390/diagnostics16040631

**Published:** 2026-02-21

**Authors:** Adnan Haider, Muhammad Arsalan, Kyungeun Cho

**Affiliations:** 1 Department of Computer Science and Artificial Intelligence, College of Advanced Convergence Engineering, Dongguk University-Seoul, 30 Pildongro 1-gil, Jung-gu, Seoul 04620, Republic of Korea; adnanhaider@dgu.ac.kr; 2 College of Engineering, Qatar University, Doha 2713, Qatar; muhammad.arsalan@qu.edu.qa

**Keywords:** artificial intelligence, medical image analysis, semantic segmentation, blastocyst segmentation, multimodal segmentation

## Abstract

**Objectives:** Global infertility rates are rapidly increasing. Assisted reproductive technologies combined with artificial intelligence are the next hope for overcoming infertility. In vitro fertilization (IVF) is gaining popularity owing to its increasing success rates. The success rate of IVF essentially depends on the assessment and inspection of blastocysts. Blastocysts can be segmented into several important compartments, and advanced and precise assessment of these compartments is strongly associated with successful pregnancies. However, currently, embryologists must manually analyze blastocysts, which is a time-consuming, subjective, and error-prone process. Several AI-based techniques, including segmentation, have been recently proposed to fill this gap. However, most existing methods rely only on raw grayscale intensity and do not perform well under challenging blastocyst image conditions, such as low contrast, similarity in textures, shape variability, and class imbalance. **Methods:** To overcome this limitation, we developed a novel and lightweight architecture, the microscopic multimodal interaction segmentation network (MiMics-Net), to accurately segment blastocyst components. MiMics-Net employs a multimodal blastocyst stem to decompose and process each frame into three modalities (photometric intensity, local textures, and directional orientation), followed by feature fusion to enhance segmentation performance. Moreover, MiMic dual-path grouped blocks have been designed, in which parallel-grouped convolutional paths are fused through point-wise convolutional layers to increase diverse learning. A lightweight refinement decoder is employed to refine and restore the spatial features while maintaining computational efficiency. Finally, semantic skip pathways are induced to transfer low- and mid-level spatial features after passing through the grouped and point-wise convolutional layers. **Results/Conclusions:** MiMics-Net was evaluated using a publicly available human blastocyst dataset and achieved a Jaccard index score of 87.9% while requiring only 0.65 million trainable parameters.

## 1. Brief Introduction

**Figure 1:**
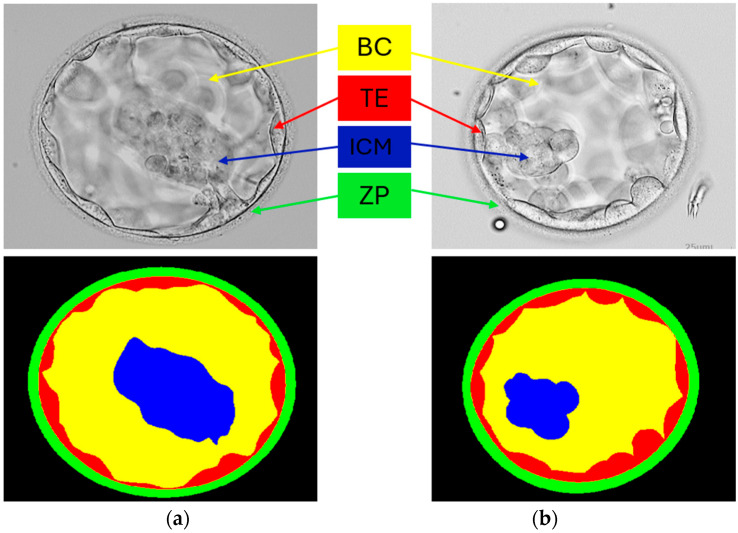
Sample images from the human blastocyst dataset with pixel-level annotation provided in row 2. (**a**) Sample image 1 and (**b**) sample image 2.

Artificial intelligence is significantly affecting the evolution of healthcare. Rising infertility rates pose a serious challenge for future generations; a recent survey reported that 80 percent of couples suffer from infertility [1]. Infertility not only inhibits reproduction but is also strongly associated with mental health problems [2]. Several developments, including assisted reproductive technology, aim to address these serious generational challenges. Among all assisted reproduction methods, in vitro fertilization (IVF) is becoming popular because of its increasing success rates [3]. In IVF, an embryologist selects suitable embryos and transfers them to the uterus for further processing. An embryo usually becomes a blastocyst on the fifth day [4]. Therefore, embryo selection is critical for the success of IVF procedures. However, embryologists typically rely on manual assessment and inspection, which is a time-consuming, resource-intensive, and subjective approach. Computer-aided methods are required to fill this gap and automate this critical process. Filho et al. [5] reported that more than one million IVF procedures per year are performed globally. Regarding success rates, almost one-third of IVF procedures have been successful in Canada [6], for example. The popularity and effectiveness of IVF are undeniable; however, some associated risks must be considered [7]. A sample image from a human blastocyst image dataset, including pixel-level annotations of the inner cell mass (ICM), blastocoel (BC), trophectoderm (TE), and zona pellucida (ZP), is shown in Figure 1.

## 2. Related Works

Over the last decade, AI-based methods have transformed the world. However, healthcare and well-being remain top priorities worldwide. Several deep learning-based methods have been developed for intelligent disease diagnosis and prognosis [8,9]; such methods are especially effective in early disease detection and analysis [10]. Several methods based on both traditional image processing and deep learning have also been proposed to better manage infertility. Wong et al. [11] used non-invasive imaging to classify and predict the developmental details of blastocysts. Similarly, another method [12] used a retinex filter, level set, and morphology-aware post-processing for TE component segmentation. Saeedi et al. [13] proposed an automatic method for identifying the TE and ICM using texture data. They used the watershed transform along with tangible map generation from the histogram to detect two components of the blastocyst.

In another study, Kheradmand et al. [14] employed the discrete cosine transform to automatically segment the ZP, TE, and ICM components. A convolutional neural network with two layers was also used to identify different features from blastocyst components. However, this study did not consider the BL class, which is an important class in the blastocyst. Rad et al. [15] modified the famous U-Net [16] architecture using a convolutional layer with higher dilation rates. They exploited parameters such as kernel size, receptive field, and network depth to optimize segmentation performance. However, they considered only the ICM class for segmentation. In [6], the same researchers extended their work to ZP detection using a cascaded depth-wise pyramid with dense upsampling. They used a patch-based approach with a trainable parameter-intensive network and focused only on the ZP component segmentation. Rad et al. [17] developed an advanced TE-specific segmentation architecture. They created a comprehensive framework in which four networks were designed to detect TE pixels, after which a dedicated multiscale architecture combined all four models and provided predictions for the TE class only.

Huang et al. [18] used time-lapse image files to analyze expanding human blastocysts. They also used the U-Net architecture; however, they detected entire blastocysts, rather than separately detecting their components. Another method proposed by Wang et al. [19] used VGG-16 to classify multifocal images. An ensemble of MobileNetV2 and VGG-16 yielded enhanced classification performance. Some of the existing methods exploited multi-modal approaches for embryonic analysis. Research work presented by Kim et al. [20] employed multimodal learning to analyze the embryonic viability. They used private data from a time-lapse video alongside the electronic health record. In the methodology, they used two processing pipelines. In the first pipeline, a vision transformer is used to deal with video and electronic health records. In the second pipeline, morphological features are extracted from the video data and then, in the next stage, fed for further processing with electronic health records. Despite using a pre-trained model, the authors express limitations of vision transformers because of limited supervised training samples and intend to refine fine-tuning in future work. Mimics-Net did not use any pretrained network and trained from scratch. Similarly, another study by Zhang et al. [21] proposed a multimodal framework for the assessment of blastocysts and evaluated their method with privately collected data. This method uses image patches and textual modalities for classification and different tasks for embryonic assessment. This study only focused on ICM and TE components. MiMics-Net provides pixel-level prediction for all components of the blastocyst. This pixel-level prediction enables the MiMics-Net to compute different ratios, such as area for further analysis.

Similarly, in a recent study carried out by Hussain et al. [22], a pixel-level segmentation architecture was used without any pooling operation for feature map size reduction. This method employs some building blocks, such as rapid, swift, and enhancement blocks for enhanced learning and boosting performance. The pooling layer is avoided to reduce the spatial loss. This method relied only on raw intensities and could not perform well, especially for both ZP and TE classes, and required 1.1 million parameters. MiMics-Net used multiple modalities and showed overall better performance using only 0.65 million parameters. Another recent research work by Miled et al. [23] exploited different attention mechanisms, such as channel, spatial, and scale attention, to improve the segmentation performance. This work employed ResNet50 as the backbone for performing the segmentation task. They. This method also relied on only raw intensity images and did not make any predictions for the BC class. MiMics-Net did not rely on any backbone or trained weights and delivered better segmentation accuracies.

### 2.1. Literature Gap

Accurate blastocyst segmentation is crucial to help in informed decision-making by the embryologist. Most existing methods rely only on raw grayscale intensity; this approach creates limitations, especially in challenging cases. Moreover, although some methods provide acceptable segmentation performance, they require a large number of trainable parameters. Therefore, to fill this gap, we propose a robust segmentation architecture capable of using three key modalities (photometric intensity, texture, and directional orientation), employing valuable building blocks and a lightweight refinement decoder to provide effective performance while requiring very few trainable parameters.

### 2.2. Motivation and Contributions

First of all, any technique or automated mechanism that can help in reducing infertility itself is a great motivation. As mentioned in the previous sections, accurate segmentation of the compartments of the blastocyst helps in viability assessment of the blastocyst. Typically, still, manual assessments are carried out to analyze the viability. Several automated methods have been proposed, but they lack robustness or require heavy architectures. The assessment and inspection of blastocysts are crucial for improving the success rate of IVF; therefore, advanced segmentation algorithms are crucial. Blastocyst images pose several challenges, such as low contrast, shape variability, similarity of features, and class imbalance, which make accurate segmentation very difficult.

The contributions of this study are summarized as follows:

- We propose a novel segmentation architecture, the microscopic multimodal interaction segmentation network (MiMics-Net), to accurately segment the components of the blastocyst image. MiMics-Net employs a multimodal blastocyst input stem (MBI stem) to decompose the input frame into three modalities—photometric intensity, texture via local binary patterns, and directional orientation through Gabor responses—to capture complementary visual properties. These multimodal features are processed using MiMic dual-path grouped blocks (MiMic-DPG blocks), followed by feature fusion via point-wise convolution.
- The MiMic-DPG blocks are based on parallel grouped convolutional paths, which aid in diverse learning, and point-wise convolution aids in cross-channel re-weighting. At the end of the decoding process, the compact bottleneck (ComB) endows the architecture with the global context of the embryo while containing the trainable parameter count.
- A lightweight refinement decoder (LRD) helps to refine and detect the boundaries of the blastocyst components without excessively increasing trainable parameters. Semantic skip pathways (SSPs) are built to transfer low- and mid-level spatial features after passing through grouped and point-wise convolutional layers. MiMics-Net was evaluated using a publicly available dataset and achieved a Jaccard index (JC) score of 87.9% while requiring only 0.65 million trainable parameters.

The remainder of this paper is organized as follows. Section 3 and Section 4 present the proposed methodology and experimental results, respectively. Section 5 discusses the findings; finally, Section 6 concludes the study.

## 3. Materials and Methods

### 3.1. Databases

In this research, we used the same publicly available dataset as in [13] and other state-of-the-art methods. To date, this dataset is the only publicly available blastocyst segmentation dataset. Since a public database was used, we only split the data according to the instructions of the dataset provider. We adjusted the training image size using the MATLAB framework [24]. This dataset contains 235 blastocyst images processed using the Hoffman method. These images were collected from various patients at the Canadian Pacific Center for Reproduction. Additionally, the images were collected between 2012 and 2016. The blastocyst images were annotated at the pixel level by expert embryologists affiliated with the dataset provider [25]. The official data-splitting protocol used in many previous studies is adopted to fairly compare with existing methods, such as [22]. A data split of 85% (200 images) for training, while 15% (35 images) for testing is used. Two example images from the dataset are presented in Figure 1.

### 3.2. Methodology

#### 3.2.1. Summary of Proposed Method

**Figure 2:**
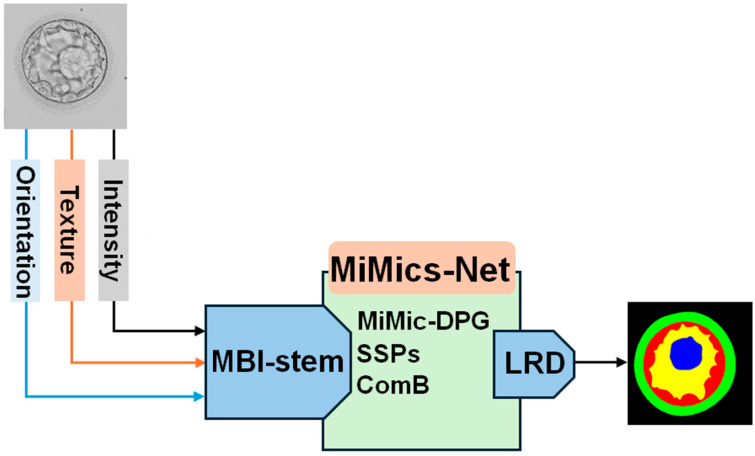
The overview of the proposed methodology (MBI-stem: multimodal blastocyst input stem; MiMic-DPG: MiMic dual-path grouped blocks; SSPs: Semantic skip pathways; ComB: Compact bottleneck; LRD: Light refinement decoder).

The morphological assessment and visual inspection of blastocysts are vital steps for successful IVF. However, most embryologists rely on manual assessments, which are tedious and subjective. As shown in Figure 2, which presents an overview of our methodology, we developed MiMics-Net to offer reliable segmentation even under challenging conditions, without requiring a large number of trainable parameters. MiMics-Net employs an MBI stem that is capable of decomposing and processing crucial modalities such as photometric intensity, texture through local binary patterns, and directional orientation via Gabor responses. The multimodal feature processing and fusion in the MBI stem help MiMics-Net to maintain high accuracy even for images with low contrast and components with similar texture. Moreover, MiMic-DPG, ComB, and the SSPs assist with diverse learning and help maintain reliable performance. Finally, the encoded features are fed into the LRD. The LRD refines the features through feature fusion and efficiently upsamples the feature map size without compromising the trainable parameters. Finally, a predicted mask is generated while labeling all components at the pixel level.

#### 3.2.2. Network Diagram of MiMics-Net and Its Functionality

**Figure 3:**
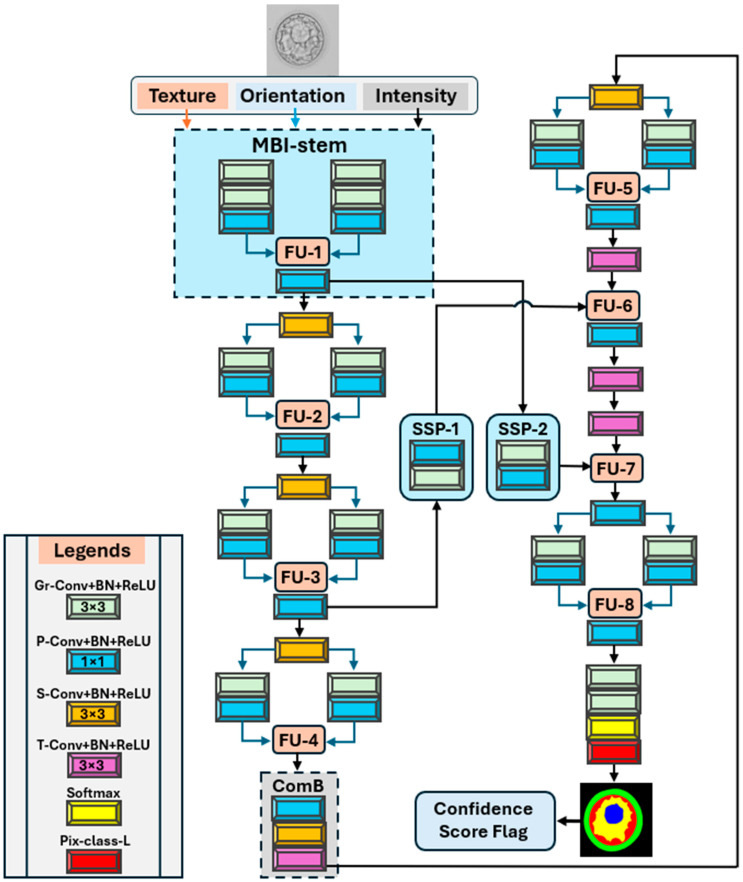
Architecture of MiMics-Net for blastocyst segmentation. (MBI-stem: multimodal blastocyst stem; FU: Feature fusion; SSP: Semantic skip pathways; ComB: Compact bottleneck; Gr-Conv: Grouped convolutional layer; BN: batch normalization layer; ReLU: Rectified linear unit; S-Conv: strided convolutional layer; T-Conv: transposed convolutional layer; P-Conv: point-wise convolutional layer; Pix-class-L: pixel classification layer).

The structural formation of blastocysts is complex, and their compartments pose serious challenges for segmentation algorithms. Relying only on raw grayscale intensities is insufficient because of the many texture- and orientation-related descriptors. To overcome these challenges, we developed MiMics-Net to segment blastocyst components using multiple modalities. A network diagram of MiMics-Net is shown in Figure 3. The MBI stem is a multichannel representation of the original blastocyst microscopy frame, where each channel models a different modality of visual information: photometric intensity, texture through local binary patterns, and directional orientation via Gabor responses. The local binary patterns help to detect micro-textural differences, for example, between ZP and TE. Gabor responses at orthogonal orientations (0° and 90°) provide orientation cues and help to detect roughly vertical or horizontal structures. Overall, the MBI stem helps distinguish boundaries, even at low contrast, and minimizes texture confusion. These multimodal features are further activated through parallel grouped convolutional layers and fed to the MiMic-DPG blocks. Each MiMic-DPG block is composed of two parallel grouped convolutional paths. Each path starts with a grouped convolutional (G-con) layer, followed by batch normalization (BN) and rectified linear unit (ReLU) layers. These parallel dual grouped paths enable the model to obtain diverse multimodal learning. The diverse multimodal features are later fused using a point-wise convolutional layer (P-con). This layer refines the group features and feeds them into a feature fusion unit (FU-1). FU-1 concatenates the multimodal features, followed by another P-con layer to further blend the modalities.

The feature map size of the fused multimodal features is reduced using the first strided convolutional (S-con) layer with a stride of 2. In the second MiMic-DPG block, the downsampled features are again split into dual grouped paths for further activation, followed by another feature fusion at FU-2. Similarly, features are further downsampled, processed, and fused (at FU-3 and 4) through two more MiMic blocks for further diverse learning through the independent learnable random weights. After FU-4, the features are activated using a ComB. In the ComB, the feature map size of 50 × 50 is further downsampled using the final S-con, followed by BN and ReLU, and the final feature map size of the architecture becomes 25 × 25. Features are not further downsampled owing to potential information loss of the minor blastocyst components. ComB is the bottleneck and bridge between encoding and decoding, and helps integrate the global context of the embryo while keeping the trainable parameters in control. The final feature map size is increased using the first transposed convolutional (T-con) layer. The upsampled spatial features are fed into the first MiMic-DPG block in the LRD.

Many existing methods require many trainable parameters. MiMics-Net is designed to exhibit reliable performance while requiring only 0.65 million trainable parameters. The LRD helps to refine and detect the boundaries of blastocyst components without requiring extensive trainable parameters. In the LRD, MiMic-DPG is used only near the maximum depth and before the final prediction for rich yet affordable feature refinement. Fused features in FU-5 are further mixed via P-con and transferred to FU-6 for further fusion. Mid-level features from the third MiMic-DPG block of the encoder are transferred through SSP-1 and fused with upsampled spatial information at FU-6. SSP-1 transfers mid-level encoder features and processes them through the G-con layer, followed by P-con after filtering and re-weighting. After mixing through the P-con layer, the fused features at FU-6 are upsampled by a pair of T-con layers, followed by BN and ReLU connected in series.

The final T-con layer (before FU-7) completely restores the feature map size to the input size (400 × 400) and feeds the features to FU-7. The rich multimodal mixed features from the MBI stem are transferred and processed through SSP-2 and fused in FU-7 (immediately before the last MiMic-DPG). These fused features from FU-7 are fed to the last MiMic-DPG block and, after processing through parallel group paths, are fed to the last fusion of the network at FU-8. After FU-8, the features are finally squeezed and activated through P-con and G-con with five channels (matching the number of classes in the blastocyst). MiMics-Net used a kernel size of 3 × 3 for all types of convolutional layers except P-con as 1 × 1. Finally, the pixel classification layer using softmax classifies the components of the blastocyst at the pixel level. In the test images, we quantify segmentation confidence using the mean softmax margin (difference between the highest and second-highest class probabilities) aggregated over all pixels. Moreover, the threshold is set as the 5th percentile of validation confidence. In our data, this corresponded to approximately 0.75. Hence, test images with confidence below the threshold are flagged for manual review.

No pretraining, weight migration, or backbone of any other architecture is employed in our architecture. The network is trained from scratch.

#### 3.2.3. Experimental Details and Data Preparation

**Figure 4:**
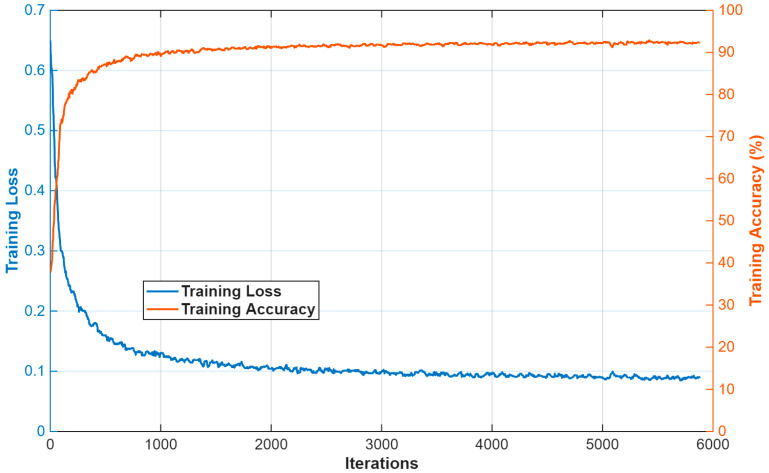
MiMics-Net training loss and accuracy plot.

**Figure 5:**
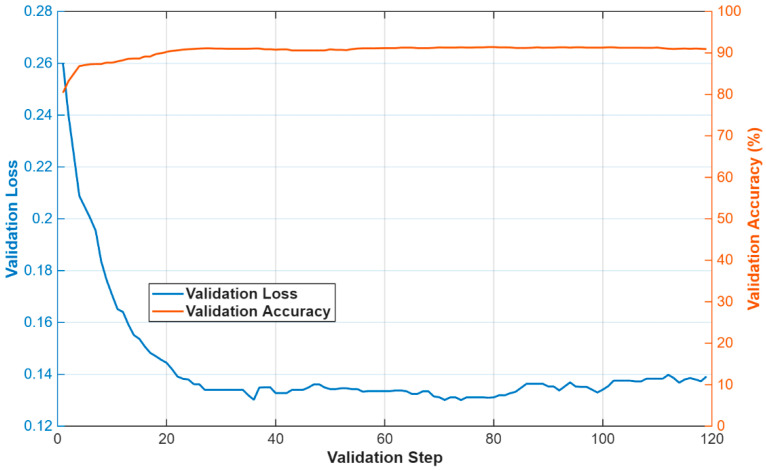
MiMics-Net validation loss and accuracy plot.

MiMics-Net was tested to segment components of the blastocyst in a challenging blastocyst database. The training process is not dependent on any kind of weight transfer or backbone. MiMics-Net is trained from scratch. Generalized dice loss [26] is used to deal with class imbalance and for effective training of the network. As shown in Figure 1, blastocyst images are partitioned into five classes. The pixels from BC and BG often substantially outnumber those of other classes that triggers the class imbalance problem. For the experiment of the current research, the Adam [27] optimizer was adopted due to its abilities of fast convergence compared with existing optimizers. In addition, a sufficient number of training images were created after augmentation for training purposes. The initial parameters for the initial learning rate (0.001), square gradient decay factor (0.95), and L2 regularization (0.0005) were determined. The training and validation loss–accuracy plots are shown in Figure 4 and Figure 5, respectively. A progressive learning with an increase in accuracy and a decrease in loss can be seen in Figure 4. In both plots, the accuracy and loss are shown in orange and blue, respectively. In the initial iteration, the network incurs a larger loss; later, the network learns the classes of blastocyst more quickly to gradually reduce the loss as the learning proceeds, identifying classes in the blastocyst. The proposed MiMics-Net was designed and applied using the MATLAB 2025a framework [24] on a computer equipped with an Intel i9-14900K CPU, 64 GB of RAM, and an NVIDIA GeForce RTX 4090 GPU.

**Figure 6:**
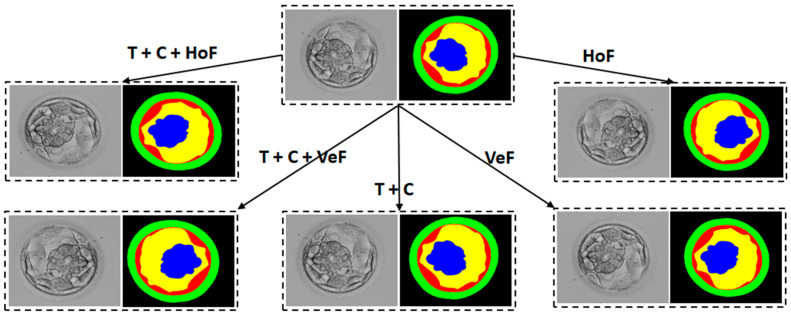
Overview diagram for the data augmentation (T: translation; HoF: horizontal flipping, VeF: vertical flipping; C: cropping).

In the experiments, the same data splits, experimental protocols, and evaluation method used by the state of the art were followed for a fair comparison. Medical images require annotations by experts; therefore, the unavailability of sufficient medical data is a widespread problem. To cover this limitation, image augmentation is employed to artificially create optimal data. In our experiments, the data augmentation included basic translation (T), horizontal flipping (HoF), vertical flipping (VeF), and cropping (C). A total of 3200 images were produced after applying augmentation. An overview of data augmentation is presented in Figure 6.

## 4. Results

### 4.1. Evaluation Measure

The trained MiMics-Net model was applied to unseen testing images. The Jaccard index (JC) and dice similarity coefficient (DSC) are widely accepted metrics for evaluating segmentation models, especially for blastocyst application [28,29]. During testing, a prediction mask is generated by MiMics-Net for each class, and the final results were calculated on the basis of the false positive (fp), true positive (tp), and false negative (fn) pixels.

(1) $$JC=\frac{tp}{tp+fp+fn}$$

### 4.2. Comparing MiMics-Net with State-of-the-Art Methods

In this subsection, the numerical results generated by MiMics-Net are compared with those of the existing methods. MiMics-Net was thoroughly tested for the segmentation of blastocyst components and generated a prediction for each class. Pixel-level prediction results were compared with standard annotations, and the resulting JC score was recorded for every class. A mean DSC is also calculated and compared with available results from existing methods. In Table 1 and Table 2, the segmentation results are computed for the ablation and comparison with existing methods, respectively. Table 1 clearly shows the contribution of the key modules, such as MBI-stem, MiMic-DPG, and SSP, in producing a final reliable accuracy. Similarly, MiMics-Net delivers a convincing score compared with existing methods while requiring a very small number of trainable parameters (0.65 million). Better semantic segmentation performance, even with trivial improvements in blastocyst components, can improve the overall visual assessment. Quantitative results also confirm the contribution of multimodal-based processing for segmenting compartments of the blastocyst. The ICM region presents the comparatively ambiguous boundaries and the highest intra-class texture variation among blastocyst compartments. It usually shares intensity and texture characteristics with adjacent blastocoel regions, making discrimination challenging. Moreover, ICM occupies a relatively small area, increasing class imbalance effects. On the other hand, transformer-based models such as SegFormer use global attention to model long-range dependencies, and MiMics-Net manages the tradeoff between lightweight deployment and performance using local multimodal features.

### 4.3. Qualitative Results Produced by MiMics-Net for Blastocyst Component Segmentation

**Figure 7:**
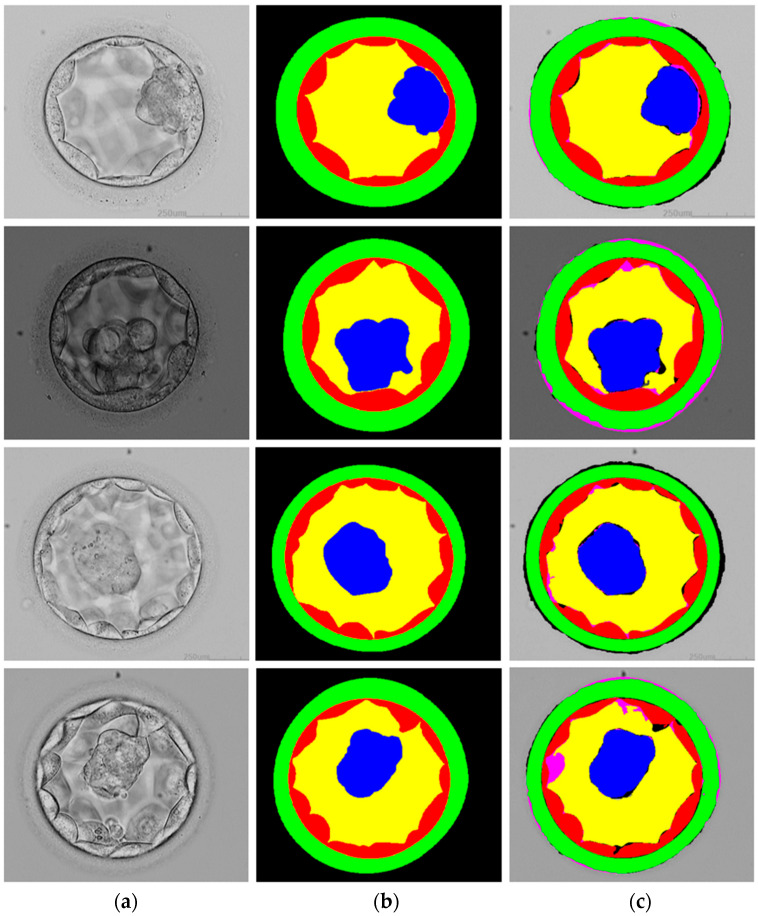
Examples of good segmentation visuals produced by MiMics-Net (**a**) Input image, (**b**) Ground truth image, and (**c**) Blastocyst image segmented by MiMics-Net. The tp pixels of TE, ICM, ZP, BL, and BG are presented in red, blue, green, yellow, and gray colors, respectively. The fp and fn pixels are presented in black and pink, respectively.

MiMics-Net uses five channels in its last layer before softmax. Each channel of the final layer refers to one of the five classes in the blastocyst image: ICM, BL, TE, ZP, and BG. The visual results generated by MiMics-Net are presented in Figure 7. The input images are presented in the first column, while the ground truth and the prediction by MiMics-Net are presented in the second and third columns, respectively. In Figure 7, the accurately predicted pixels (tp) of TE, ICM, ZP, BL, and BG are presented in red, blue, green, yellow, and gray (the same colors as in the ground truth image), respectively. In the same way, the fp pixels are shown in black, whereas fn pixels are shown in pink. Overall, the predicted visuals reinforce and validate the strength of MiMics-Net.

**Figure 8:**
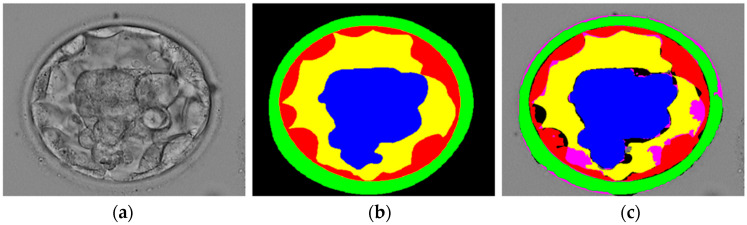
Example of compromised segmentation visuals produced by MiMics-Net. (**a**) Input image, (**b**) Ground truth image, and (**c**) Blastocyst image segmented by MiMics-Net. The tp pixels of TE, ICM, ZP, BL, and BG are presented in red, blue, green, yellow, and gray colors, respectively. The fp and fn pixels are presented in black and pink, respectively.

Similarly, an example image showing relatively compromised segmentation performed by MiMics-Net is shown in Figure 8. This compromised segmentation performance can be attributed to the complex and similar structures of components, which have indistinct boundaries in certain areas. Nevertheless, MiMics-Net still provided competitive performance. The local binary pattern/Gabor can be less informative when the texture is weak or when illumination makes edges misleading; therefore, failure is likely associated with weak local texture and ambiguous oriented edges under poor illumination, reducing the usefulness of texture-enhanced cues.

## 5. Discussion

Embryologists assess embryo viability based on visual and morphological assessments, which emphasize the importance of the precise segmentation of blastocyst components. Many researchers acknowledge the positive role of the morphological assessment of blastocysts in this process [33,34]. These semantic segmentations of the blastocyst compartments enable embryologists to perform computational and morphological analyses. Many researchers have considered only a single component, limiting the scope of the analysis. MiMics-Net considers all blastocyst component classes (ZP, ICM, BL, TE, and BG) and makes multiclass predictions to enable thorough visual or computational representations.

According to Arsalan et al. [28] the areas and locations of blastocyst components are important for embryo viability and assessment. The predicted semantic segmentation mask can also be used to compute the areas of different components for computational analysis. Similarly, the locations of segmented components can further enrich the analysis. The BC component is considered to be one of the most important components in the context of decision-making by embryologists. Therefore, accurate segmentation of BC components can assist embryologists in making informed decisions. Additionally, Bori et al. [35] referred to the number of pixels in each component of the blastocyst and the corresponding areas as important for the assessment of the embryo. The results obtained using MiMics-Net show that the proposed method can provide a reliable area, position, and number of pixels for each component of the blastocyst.

The structure of the blastocyst is complex from a computer vision perspective. Therefore, it is important and beneficial to use multiple modalities to maintain satisfactory performance, even when dealing with challenging cases. Moreover, trainable parameters are important factors for computational efficiency. Because segmentation prediction is performed at the pixel level, a robust architecture capable of delivering reliable performance with a small number of trainable parameters is required. MiMics-Net fulfills all of these requirements by decomposing, processing, and fusing multiple modalities (intensity, texture, and orientation), thereby achieving competitive performance while requiring only 0.65 million trainable parameters. Hence, the proposed method can assist embryologists by providing visual or computational assistance. The effect of blur and noise on the performance of MiMics-Net was analyzed in Table 3 to assess the robustness of the proposed method. Gaussian noise with variance values of 0.0005, 0.001, and 0.002, whereas salt and pepper noise with noise densities of 0.75% and 1.5% are applied. Similarly, blurring with sigma values of 0.5 and 1 is also applied. Considering the small spatial dimensions and similarities among components, MiMics-Net still delivers satisfactory performance with noises and blur. MiMics-Net is not tied to a specific microscope model or optical pipeline. The input representation combines intensity, local texture encoding, and orientation-selective responses, which are contrast and structure-driven rather than device-specific. These descriptors are known to be more stable across illumination and acquisition settings compared to raw intensity alone. Furthermore, geometric augmentation was applied during training to reduce sensitivity to spatial and photometric variation. The segmentation performance of MiMics-Net remained stable under moderate perturbations, signifying that the proposed texture-enhanced input and lightweight encoder–decoder are not narrowly tuned to a single photometric setting; nevertheless, prospective evaluation on modern time-lapse platforms remains essential and will be considered in future work

Blastocysts exhibit substantial morphological variability, including different expansion stages, partial collapse, and hatching. These conditions often reduce boundary clarity between compartments. MiMics-Net addresses this through multi-scale encoding and dual-path feature extraction blocks that capture both coarse structural context and fine texture cues. The multimodal input channels further enhance weak boundary visibility. However, as illustrated in the compromised example (Figure 8), segmentation accuracy can decrease in collapsed or irregular embryos, which can be accounted for as a limitation. Despite its numerous merits and achievements, MiMics-Net has certain limitations. The method benefits from augmentation to compensate for the limited dataset size, which indicates that access to larger annotated datasets could further improve robustness. Second, the dataset used here is the only dataset available with component-wise pixel-level annotations. Therefore, we intend to collect our own dataset in the future.

We acknowledge that the varying degrees of expansion or collapse of embryos can be challenging for any model. However, the dataset does not contain information/data from different stages. Still, in Section 4, we applied blur and noise to make the boundaries of the blastocyst’s components relatively unclear and evaluated MiMics-Net. The proposed method maintained satisfactory performance despite moderate blur and noise effects.

## 6. Conclusions

Automatic morphological assessment of blastocysts can assist embryologists in viability analysis. We developed a novel segmentation architecture, MiMics-Net, to accurately segment the components of the blastocyst image. MiMics-Net employs a multimodal blastocyst input stem (MBI stem) to decompose the input frame into three modalities—photometric intensity, texture via local binary patterns, and directional orientation cues through Gabor responses—to capture complementary visual properties for accurate segmentation. The proposed method employs valuable modules such as MiMic-DPG blocks, SSPs, and an LRD to maintain reliable segmentation performance, while requiring a very small number of trainable parameters. The proposed method achieved a JC score of 87.9% while requiring only 0.65 million trainable parameters. Overall, MiMics-Net provides an accessible and computationally efficient framework for blastocyst compartment segmentation, potentially supporting downstream automated embryo quality assessment for assisted reproductive technology.

### Future Work

This study evaluates MiMics-Net on a publicly available benchmark dataset. Although augmentation improves variability tolerance, true robustness requires multi-center validation across different microscopes and acquisition protocols. Such evaluation is planned in future work. Moreover, a comparison of Gardner grades (or similar) derived from MiMics-Net segmentations versus those from raw images can demonstrate whether the model preserves the morphological features essential for clinical decision-making. However, this requires expert embryologist grading labels that are not included in the public dataset, and it can also be accounted for in future work.

## Figures and Tables

**Table 1:** Ablation studies for blastocyst segmentation using MiMics-Net (Results in %). (mJc: mean Jaccard index; The symbol “√” shows inclusion of the respective module in the network).

MBI-Stem	MiMic-DPG	SSPs	mJC
			82.55
√	√		86.38
	√		83.83
√		√	86.16
	√	√	85.91
√	√	√	87.9

**Table 2:** Comparing quantitative results of MiMics-Net with state-of-the-art methods. (M represents million, mJC: mean Jaccard index, Mean DSC: mean dice similarity coefficient, Train. Para: trainable parameters, “-” is used where information is not available).

Methodology	Epochs	Train. Para (M)	ZP	TE	BL	ICM	BG	Mean DSC	Mean JC
DeepLab V3 [29]	-	40	0.806	0.7398	0.8084	0.7835	0.9449	-	0.8165
FCN [25]	-	134	0.765	-	-	-	-	-	-
Blast-Net [6]	-	25	0.8107	0.7652	0.8115	0.8079	0.9474	-	0.8285
Ternaus-Net [30]	-	10	0.7758	0.7616	0.8024	0.7861	0.945	-	0.8142
PSP-Net [31]	-	35	0.7828	0.7483	0.8057	0.7926	0.946	-	0.8151
SSS-Net Dense [28]	-	4.04	0.845	0.7815	0.8451	0.8868	0.9582	-	0.8634
SSS-Net Residual [28]	-	4.04	0.8494	0.774	0.8288	0.8839	0.9603	-	0.8593
U-Net [16]	-	31.03	0.7903	0.7506	0.7932	0.7941	0.9404	-	0.8137
ECS-Net [4]	-	2.83	0.8526	0.7843	0.8534	0.8841	0.9487	-	0.8646
SegFormer [32]	-	27.35	0.8995	0.7656	0.8075	0.9049	0.9242	-	0.8604
PFRS-Net_Plain [22]	50	1.0	0.8127	0.7812	0.8462	0.8751	0.9587	0.9166	0.8547
PFRS-Net_Final [22]	50	1.1	0.8547	0.7929	0.8522	0.8859	0.9597	0.9278	0.8691
MiMics-Net (proposed)	35	0.65	0.8538	0.8265	0.9085	0.8497	0.9601	0.9343	0.879

**Table 3:** Effect of blur and noise on segmentation performance of Mimics-Net (Results in %) (mJc: mean Jaccard index).

Method	mJC
MiMics-Net (Salt&pepper noise, density = 0.75%)	86.52
MiMics-Net (Salt&pepper noise, density = 1.5%)	78.79
MiMics-Net (Gaussian noise_variance = 0.0005)	88.24
MiMics-Net (Gaussian noise_variance = 0.001)	86.97
MiMics-Net (Gaussian noise_variance = 0.002)	79.0
MiMics-Net (Blur, sigma = 0.5)	87.49
MiMics-Net (Blur, sigma = 1)	83.05

## Data Availability

A publicly available data set is used in this research. The original data presented in the study is openly available via the following links as follows: “https://vault.sfu.ca/index.php/s/066vGJfviJMYuP6/authenticate/showshare (accessed on 5 November 2025)” (for the password please contact psaeedi@sfu.ca). The same dataset link is also available via the publication below: https://ieeexplore.ieee.org/document/8059868 (accessed on 5 November 2025).
